# Mild Hypothermia Attenuates Mitochondrial Oxidative Stress by Protecting Respiratory Enzymes and Upregulating MnSOD in a Pig Model of Cardiac Arrest

**DOI:** 10.1371/journal.pone.0035313

**Published:** 2012-04-20

**Authors:** Ping Gong, Chun-Sheng Li, Rong Hua, Hong Zhao, Zi-Ren Tang, Xue Mei, Ming-Yue Zhang, Juan Cui

**Affiliations:** Department of Emergency, Beijing Chaoyang Hospital, Capital Medical University, Beijing, People's Republic of China; Heart Center Munich, Germany

## Abstract

Mild hypothermia is the only effective treatment confirmed clinically to improve neurological outcomes for comatose patients with cardiac arrest. However, the underlying mechanism is not fully elucidated. In this study, our aim was to determine the effect of mild hypothermia on mitochondrial oxidative stress in the cerebral cortex. We intravascularly induced mild hypothermia (33°C), maintained this temperature for 12 h, and actively rewarmed in the inbred Chinese Wuzhishan minipigs successfully resuscitated after 8 min of untreated ventricular fibrillation. Cerebral samples were collected at 24 and 72 h following return of spontaneous circulation (ROSC). We found that mitochondrial malondialdehyde and protein carbonyl levels were significantly increased in the cerebral cortex in normothermic pigs even at 24 h after ROSC, whereas mild hypothermia attenuated this increase. Moreover, mild hypothermia attenuated the decrease in Complex I and Complex III (i.e., major sites of reactive oxygen species production) activities of the mitochondrial respiratory chain and increased antioxidant enzyme manganese superoxide dismutase (MnSOD) activity. This increase in MnSOD activity was consistent with the upregulation of nuclear factor erythroid 2-related factor 2 (Nrf2) mRNA and protein expressions, and with the increase of Nrf2 nuclear translocation in normothermic pigs at 24 and 72 h following ROSC, whereas mild hypothermia enhanced these tendencies. Thus, our findings indicate that mild hypothermia attenuates mitochondrial oxidative stress in the cerebral cortex, which may be associated with reduced impairment of mitochondrial respiratory chain enzymes, and enhancement of MnSOD activity and expression via Nrf2 activation.

## Introduction

Recently, despite improvements in resuscitation techniques, the survival rate in patients with cardiac arrest has not improved [1], [2], [3], and adverse neurological outcomes remain a leading problem following return of spontaneous circulation (ROSC), which is closely related to a high post-resuscitative mortality and poor quality of life [4], [5]. One reason for these poor outcomes is a lack of neuroprotective medications capable of ameliorating ischemia/reperfusion (I/R) injury during the post-resuscitation period. However, recently, an effective treatment that has been confirmed clinically to improve neurological outcomes is mild hypothermia [6], [7], [8].

To data, the maximum therapeutic effect and mechanism of action of mild hypothermia remains unclear. This may be due to the complex mechanisms involved during global brain I/R injury when triggered by cardiac arrest and resuscitation. Increasing evidence has shown that the pathogenesis of post-resuscitation brain injury is complicated by a complex cascade of processes such as oxidative stress, excitotoxicity, disrupted calcium homeostasis, pathological protease cascades and activation of cell death signaling pathways, which are activated within minutes to hours after injury, and continue for up to 72 h or longer [4]. These processes are temperature dependent – i.e. may increase during fever and can be inhibited by mild hypothermia [4].

Mitochondrial dysfunction and oxidative stress are considered to be key determinants with respect to the extent of injury during cerebral ischemia [9]. Impairment of mitochondrial function leads to reduced ATP production, impaired calcium buffering and, in particular, the overproduction of reactive oxygen species (ROS). Under physiologic conditions, ROS do not cause injury because they are quickly scavenged by the intramitochondrial antioxidant system, which includes antioxidants such as glutathione, and antioxidant enzymes such as manganese superoxide dismutase (MnSOD) and catalase, which are regulated by nuclear factor erythroid 2-related factor 2 (Nrf2), a key nuclear transcription factor in maintaining redox balance [10]. Under pathologic conditions such as cerebral I/R, ROS excessively produce, and react with nitric oxide (NO) to produce nitrogen species (RNS). The products exceed the scavenging capacity of the endogenous antioxidant system in mitochondria, and consequently oxidative stress occurs. As a result, the excess production of mitochondrial ROS and RNS can damage mitochondria by initiating peroxidation of intramitochondrial lipids and proteins, inhibiting the activity of mitochondrial respiratory enzymes and breaking mitochondrial DNA [11], which induces cell apoptosis or necrosis [9].

**Figure 1:**
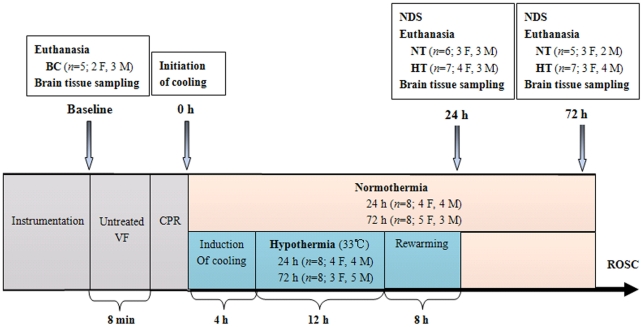
Experimental procedure. HT, mild hypothermia group; NT, normothermia group; BC, blank control group; VF, ventricular fibrillation; CPR, cardiopulmonary resuscitation; ROSC, restoration of spontaneous circulation; NDS, neurologic deficit scores; F, female; M, male.

Animal and patient studies have indicated that the protective effect of mild hypothermia against brain injury may be due to a reduction in brain metabolism, inhibition of excitatory amino acid release, attenuation of the immune response, or modification of cell death signaling pathways [4], [8], [12], [13], [14], [15]. However, the effect of mild hypothermia on mitochondrial oxidative stress and the exact mechanisms involved in cardiac arrest patients remain unknown.

In the present study, we employed a swine model of cardiac arrest to test the hypothesis that whole-body mild hypothermia attenuates mitochondrial oxidative stress in cerebral cortex by reducing impairment of mitochondrial respiratory chain enzymes and enhancing the activity and expression of MnSOD via Nrf2 activation following ROSC.

## Materials and Methods

### Animal preparation

This study was carried out in strict accordance with the guideline for animal care and use established by the Capital Medical University Animal Care and Use Committee. The protocol was approved by the Committee on the Ethics of Animal Experiments of Capital Medical University (Permit Number:2010-D-013). All surgery was performed under anesthesia and analgesia, and all efforts were made to minimize suffering.

Twenty-one male and 16 female, inbred Chinese Wuzhishan minipigs [Permit Number: SYXK (Beijing) 2008-0007, the Institute of Animal Sciences, Chinese Academy of Agricultural Sciences, Beijing, China] aged 4–6 months, weighing 24.5±1.7 kg, were fasted overnight, but had free access to water. Our choice for the Wuzhishan minipig is due to their characteristics similar to human beings in the histologic structures and physiology, and especially due to the highest inbreeding coefficient (more than 0.965), stable heredity and little variability between individual animals after 20 generations of inbreeding [16]. Thus, this sort of inbred minipig is considered as more appropriate experimental animal for cardiac arrest.

The animals were premedicated with an intramuscular injection of ketamine (20 mg/kg), followed by cannulation of an ear vein and intravenous administration of propofol (2 mg/kg) for endotracheal intubation 10 min later. Anesthesia was maintained by injection of sodium pentobarbital (8 mg/kg/h) and fentanyl (5 µg/kg/h). After premedication, pigs were supinely secured on the operating table and given normal saline (10 mL/kg/h) through a vein to maintain a central venous pressure of 5–12 mmHg. After endotracheal intubation, the animals were ventilated with a volume-controlled ventilator (Servo 900c, Siemens, Munich, Germany) with a tidal volume of 15 mL/kg, FiO_2_ of 0.21, and ventilation rate of 12 to 20 breaths/min. End-tidal PCO_2_ was monitored with an in-line infrared capnograph placed in the airway. Ventilation rate and tidal volume were adjusted to maintain normocapnia (35–45 mmHg). Arterial blood gases (ABL80, Radiometer, Copenhagen, Denmark) were analyzed to confirm adequate baseline ventilation.

The thorax skin was shaved to secure standard lead II electrocardiogram surface electrodes. To measure aortic pressure, a fluid filled catheter was advanced from the left femoral artery into the thoracic aorta. To measure right atrial pressure, pulmonary artery wedge pressure and cardiac output, a Swan-Ganz catheter (7-Fr, Edwards Life Sciences, Irvine, California, USA) was advanced from the left femoral vein and flow-directed into the pulmonary artery. The Electrocardiograph (ECG), the aortic, right atrial and pulmonary artery wedge pressure, and cardiac output were monitored continuously throughout the experiment with a monitor (Vigilance II, Edwards Life Sciences, Irvine, California, USA). To induce ventricular fibrillation (VF), a 5-Fr pacing catheter was advanced from the right femoral vein into the right ventricle. To cool pigs, a central venous catheter (Icy^TM^, Alsius Corp., Irvine, CA, USA) was advanced from the right external jugular vein into the superior vena cava. A temperature sensing Foley catheter (Integral Medical Products Co., Ltd, Shaoxing, China) was inserted into the bladder following fistulation. The central venous catheter and the temperature sensing Foley catheter were connected to an external cooling device (CoolGard 3000 system, Alsius Corp., Irvine, CA, USA). The operation was performed using aseptic surgical techniques. All catheters were calibrated before use, and unfractionated heparin (100 U/kg) was administered to prevent the catheter from clotting.

**Table 1:** Experimental conditions.

	Baseline	After ROSC		
		12 h	24 h	72 h
Weight (kg)				
NT	26.4±1.7	26.4±1.7	25.6±1.7	25.1±1.3
HT	26.6±1.8	26.4±1.7	25.6±2.2	24.8±2.0
Temperature (bladder, °C)				
NT	37.1±0.7	38.0±0.6	38.8±0.6	37.4±0.8
HT	37.0±0.6	33.1±0.3*	37.6±0.4	37.7±0.9
Heart rate (bpm)				
NT	93.8±8.5	95.4±6.8	97.8±8.4	94.3±4.6
HT	94.3±9.1	85.6±2.7*	95.4±6.7	93.8±5.3
MAP (mmHg)				
NT	111.9±7.6	108.6±5.8	107.6±8.9	109.4±9.3
HT	112.3±5.9	105.5±10.2	106.3±7.8	108.2±7.7
CO( L/min)				
NT	4.4±0.4	3.3±0.3	3.6±0.2	4.1±0.3
HT	4.3±0.3	2.5±0.2*	3.9±0.2	4.3±0.4
PAWP (mmHg)				
NT	11.1±0.5	11.0±0.4	11.3±0.6	11.0±0.5
HT	10.9±0.7	10.4±0.5	10.7±0.6	10.9±0.5
CPP (mmHg)				
NT	75.8±2.7	73.9±2.5	74.6±1.7	75.5±2.1
HT	76.5±2.9	70.8±1.9	75.2±1.9	76.3±1.8
Arterial lactate (mg/dl)				
NT	1.34±0.41	1.93±0.54	1.76±0.41	1.42±0.29
HT	1.30±0.61	1.70±0.48	1.54±0.39	1.33±0.42
Arterial PO_2_ (mmHg)				
NT	91.2±8.7	101.4±9.5	100.7±7.8	89.1±6.9
HT	91.7±6.9	103.2±7.7	101.4±6.5	90.6±8.1

Values are mean ± SD. *p<0.01 *vs*. NT group 12 h after ROSC. HT, mild hypothermia group; NT, normothermia group; BC, blank control group; ROSC, restoration of spontaneous circulation; bpm, beats per minute; MAP, mean aortic pressure; CO, cardiac output; PAWP, pulmonary artery wedge pressure; CPP, coronary perfusion pressure.

### Experimental protocol

After instrumentation, thirty minutes were allowed for hemodynamic stabilization. During the period, intra-bladder temperature was adjusted to 37°C using a heating lamp and warm packs, or an electric fan and ice bags. VF was induced by programmed electric stimulation through the fibrillation catheter, and was confirmed by the VF wave in the ECG and sharply decreased blood pressure (Figure 1). Once VF occurred, mechanical ventilation was discontinued. After 8 min of untreated VF, CPR was started with chest compressions performed by the same investigator, an experienced CPR technician from our laboratory. Meanwhile, ventilation was conducted using a bag respirator attached to the endotracheal tube with room air, and the compression-to-ventilation ratio was 30∶2. After 2 min of CPR, a single 150 J biphasic electrical shock was attempted with a Smart Biphasic defibrillator (Philips Medical Systems, Andover, MA, USA). If VF still persisted, another 2-min of CPR was resumed, followed by the first bolus of epinephrine (30 µg/kg) via the femoral vein. Additional doses of epinephrine were administered, if needed, every 3 min until ROSC was achieved. Two hundred J was used for the second and all subsequent attempts. We defined ROSC as an organized cardiac rhythm with a mean aortic pressure of greater than 60 mmHg, continuously sustained for at least 10 min or more. Resuscitation procedures were terminated if animals had no ROSC after 20 min of CPR.

**Figure 2:**
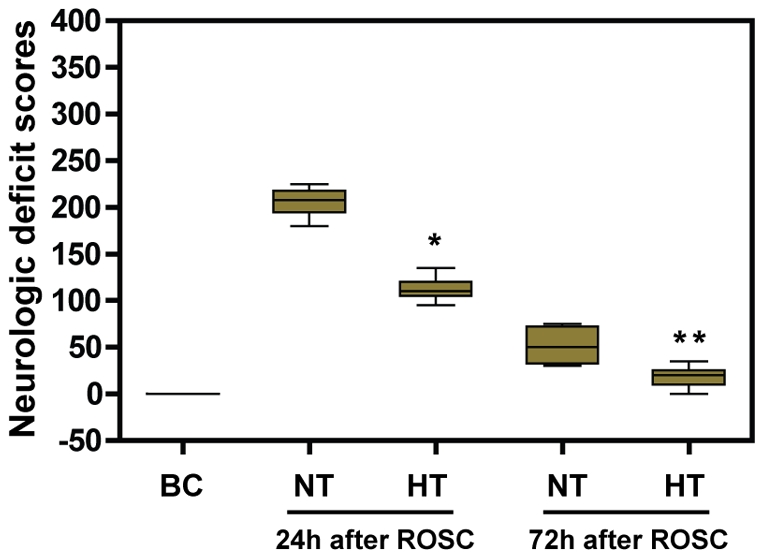
Neurologic deficit scores. 0 represents for normal and 400 for brain death. Values are given as median ± interquartile range. *p<0.01 *vs*.NT-24 h group; **p<0.01 *vs.* NT-72 h group. HT, mild hypothermia group; NT, normothermia group; BC, blank control group; ROSC, restoration of spontaneous circulation.

After ROSC, pigs were randomized into mild hypothermia group (HT, *n* = 16), or normothermia group (NT, *n* = 16) subjected to the same treatment as mild hypothermia group with the exception to no cooling. Another 5 pigs were for the blank control group (BC, *n* = 5), which only received the same anesthesia and surgery as other groups without induced VF and mild hypothermia. Pigs in the normothermic and mild hypothermic groups were again randomly assigned to subgroups on the basis of the time of euthanasia, namely normothermia 24 h after ROSC (NT-24 h, *n* = 8), mild hypothermia 24 h after ROSC (HT-24 h, *n* = 8), normothermia 72 h after ROSC (NT-72 h, *n* = 8), and mild hypothermia 72 h after ROSC (HT-72 h, *n* = 8; Figure 1).

Immediately after ROSC, mechanical ventilation was resumed with the same setting as the one used before the induction of VF. Meanwhile, according to the landmark study by Bernard et al.[7] animals were actively cooled to a target body temperature of 33°C (1.0°C/h), maintained at this temperature for 12 h, and then actively rewarmed (0.5°C/h) to 37°C using a CoolGard 3000 system. During the induction and maintenance of mild hypothermia, all animals received pancuronium bromide (0.1 mg/kg) IV to prevent shivering and muscle movement. Administration was repeated if needed.

**Figure 3:**
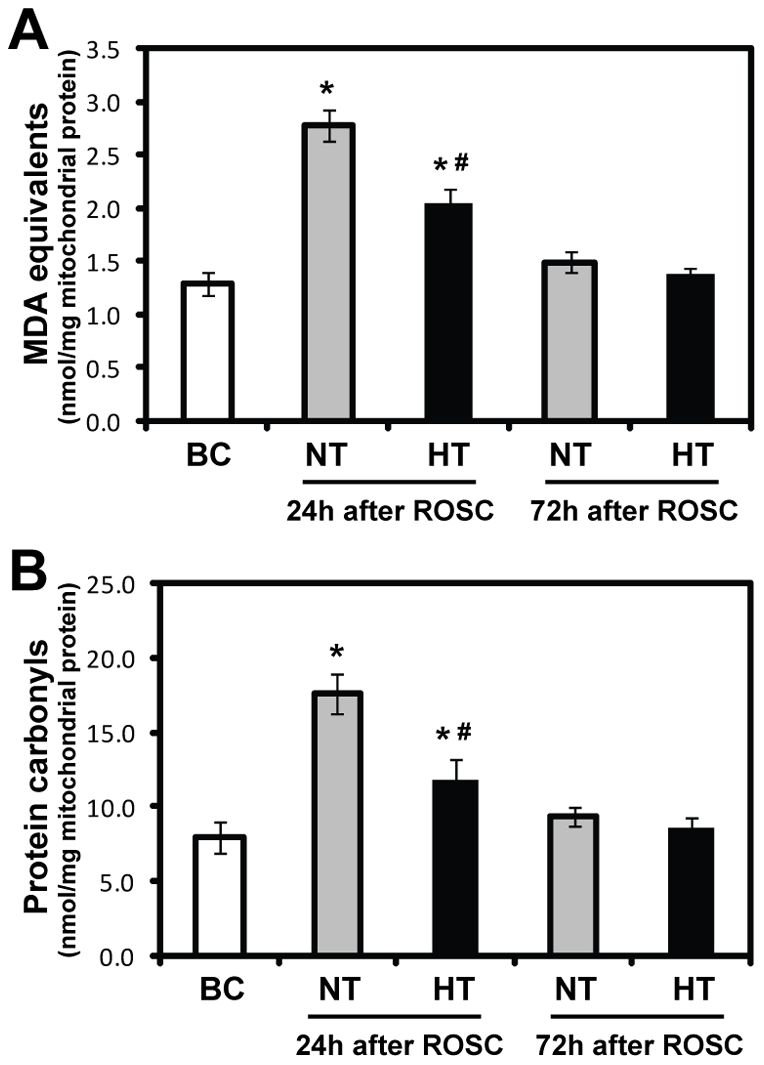
Malondialdehyde (MDA) (A) and protein carbonyls (B) in mitochondria isolated from the swine frontal cortex. Results are expressed as nmol/mg mitochondrial protein (mean ± SD). *p<0.05 *vs*. BC group; #*p*<0.01 *vs*. NT-24 h group. HT, mild hypothermia group; NT, normothermia group; BC, blank control group; ROSC, restoration of spontaneous circulation.

After ROSC, pigs received standardized post-resuscitative intensive care till the end of the rewarming phase, and then were returned into their cages. The room temperature was maintained between 20°C and 24°C. Hemodynamics were obtained hourly during the observation period. At 24 or 72 h after ROSC, animals were euthanized by injecting propofol (3 mg/kg) and then 10 mL of potassium chloride (10 mol/L). The brain was immediately removed by craniotomy and divided by a mid-sagittal cut. The right hemisphere was dissected, and the hippocampus and a portion of the precentral gyrus of the frontal lobe were fixed in 4% buffered formalin for hematoxylin-eosin staining. Another portion of the precentral gyrus of the frontal lobe was immediately collected on ice for morphological examination by electron microscopy. From the left hemisphere, frontal cortex samples (1–2 g) were excised, and the mitochondria were rapidly isolated to test respiratory enzyme activity. The remaining mitochondria and frontal cortex samples were snap-frozen in liquid nitrogen, and stored at −80°C until used. These cerebral areas were chosen because the cerebral cortex is closely related to functional outcomes, and because the CA1 region is selectively vulnerable to global cerebral ischemia in animal and human studies.

**Figure 4:**
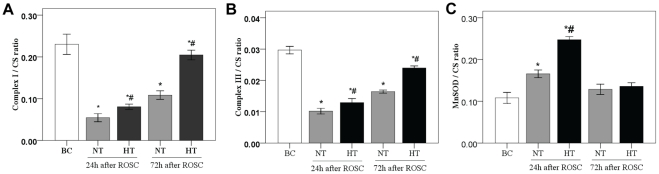
Activities of respiratory enzymes and MnSOD in mitochondria isolated from the swine frontal cortex. Respiratory enzyme (A and B) and MnSOD (C) activities are expressed as a ratio of citrate synthase activity (mean ± SD). *p<0.05 *vs*. BC group; #*p*<0.01 *vs*. NT group. HT, mild hypothermia group; NT, normothermia group; BC, blank control group; ROSC, restoration of spontaneous circulation. CS, citrate synthase.

### Isolation of mitochondria

Mitochondria were isolated from pig brain cortex according to the protocol described previously [17] with minor modifications. Briefly, before mitochondrial isolation, all solutions were cooled below 0°C until the slight appearance of ice. Brain cortex tissues from pigs were rapidly removed, immediately put into ice-cold isolation medium (225 mmol/L mannitol, 75 mmol/L sucrose, 5 mmol/L HEPES, 1.0 mmol/L EGTA, 1 mg/mL defatted bovine serum albumin, pH = 7.4) and shaken to wash out blood. Brain tissues were minced on ice in 10 mL of ice-cold isolation medium containing 0.05% (w/v) nagarse, and then manually homogenized using a glass homogenizer. Thereafter, the homogenate was added to 20 mL of ice-cold isolation medium and centrifuged at 2,000×*g* at 4°C for 4 min. After centrifugation, the supernatant was filtered using cheesecloth and centrifuged again at 12,000×g at 4°C for 9 min. To permeabilize synaptosomes, the resulting pellet was dissolved with 10 mL of ice-cold isolation medium containing 0.02% (w/v) digitonin, transferred to a small glass homogenizer, and then homogenized manually. Finally, the resultant homogenate was centrifuged at 12,000×*g* at 4°C for 11 min. After discarding the supernatant, the final pellet was resuspended in 300 µL ice-cold isolation medium (20 mg protein/mL) and kept on ice for further experimental assays. Mitochondrial protein was determined using the Bradford method.

**Figure 5:**
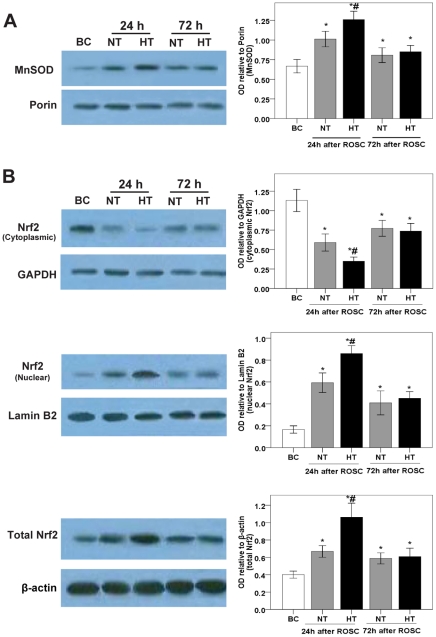
Expressions of MnSOD, and cytoplasmic, nuclear and total Nrf2 protein in the swine frontal cortex. The expressions of MnSOD (A) and Nrf2 (B) protein were measured by western blotting (*left*) using appropriate antibodies in the BC (*n* = 5), NT-24 h (*n* = 6), HT-24 h (*n* = 7), NT-72 h (*n* = 5) and HT-72 h (*n* = 7) groups. The OD of each band was measured using Image J software, and the values of MnSOD, and cytoplasmic, nuclear and total Nrf2 protein were normalized to Porin, GAPDH, Lamin B2 and β-actin, respectively (*right*). Data are expressed as the mean ± SD. *p<0.05 *vs*. BC group; #*p*<0.05 *vs*. NT-24 h group. HT, mild hypothermia group; NT, normothermia group; BC, blank control group; ROSC, restoration of spontaneous circulation; OD, optical density.

**Figure 6:**
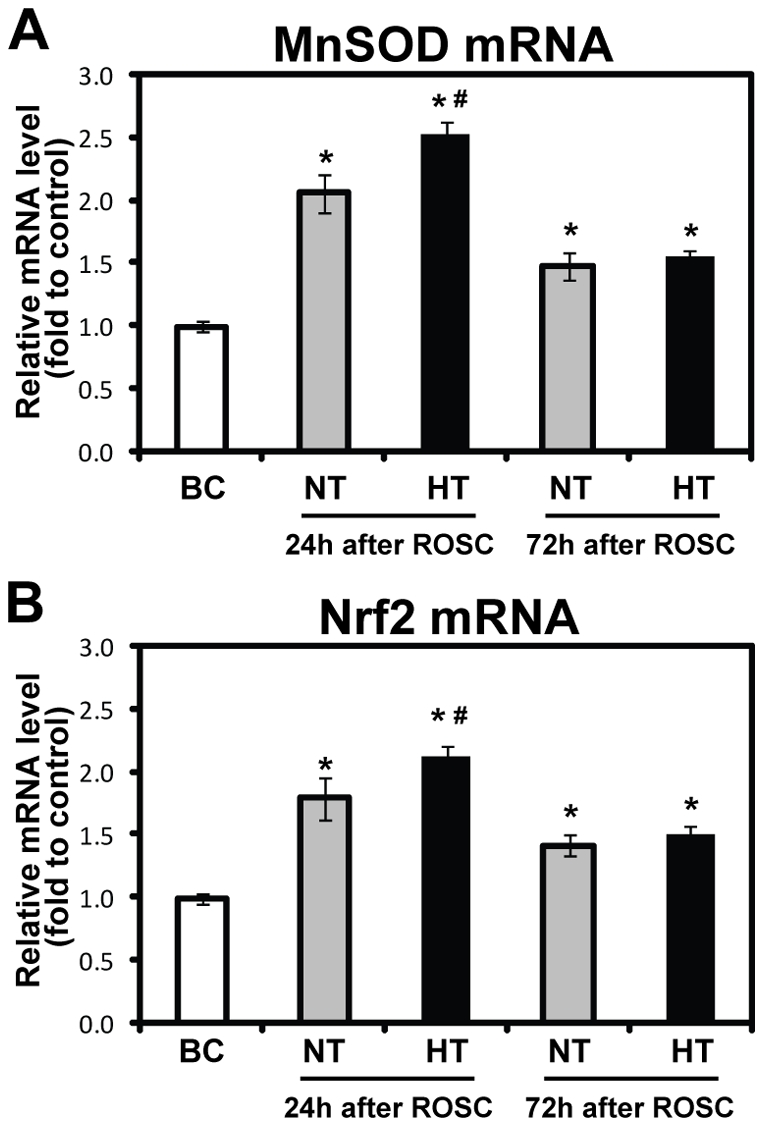
Effects of mild hypothermia on Nrf2 and MnSOD mRNA expressions in the swine frontal cortex. The mRNA levels for MnSOD (A) and Nrf2 (B) were quantified with real-time PCR. The data were normalized to β-actin. The fold changes of Nrf2 and MnSOD mRNA were calculated relative to the BC group, and results are presented as the mean fold of the blank control ± SD (*n* = 5, 6 or 7 animals for each group). *p<0.05 vs. BC group; #p<0.05 *vs*. NT-24 h group. HT, mild hypothermia group; NT, normothermia group; BC, blank control group; ROSC, restoration of spontaneous circulation.

### Determination of thiobarbituric acid reactive species (TBARS) in mitochondria

Oxidative damage to biomembranes is characterized by lipid peroxidation, which can be measured by the formation of TBARS during an acid-heating reaction [18]. The concentration of TBARS is expressed as malondialdehyde (MDA) production. To determine the status of mitochondrial oxidative stress, we measured the levels of MDA in mitochondria using a commercial MDA kit (GENMED, Shanghai, China), according to the manufacturer's instructions. Results were expressed as MDA equivalents (nmol/mg mitochondrial protein). Each sample was tested in triplicate.

### Determination of protein carbonyls in mitochondria

Oxidative damage to protein is characterized by protein carbonylation, measured based on the reaction with dinitrophenylhydrazine. To determine the status of mitochondrial oxidative stress, we measured the levels of protein carbonyls in mitochondria using a commercial protein carbonyls kit (GENMED, Shanghai, China), according to the manufacturer's instructions. The concentration of protein carbonyls was expressed as nmol/mg mitochondrial protein. Each sample was tested in triplicate.

**Figure 7:**
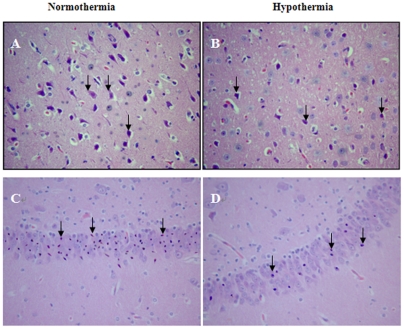
Representative microscopic changes in the cortex and hippocampus. Hematoxylin and eosin stained sections (×200), in the precentral gyrus of the frontal lobe (A and B) and CA1 area of the hippocampus (C and D) at 24 h following ROSC, showed changes in damaged neurons, including an eosinophilic cytoplasm, loss of Nissl substance and nuclear pyknosis (arrows). The number of damaged neurons from hypothermic pigs (B and D) was markedly decreased when compared to normothermic pigs (A and C).

### Assay for citrate synthase and MnSOD enzymatic activity

Citrate synthase is widely used as a mitochondrial marker because its activity [19] and mRNA [20] are constitutively expressed and do not change with age or pathological condition. The assay for citrate synthase enzymatic activity was performed spectrophotometrically as described previously [21], [22], [23]. MnSOD activity was measured spectrophotometrically at 550 nm using the MnSOD activity commercial kit (Nanjing Jiancheng Bioengineering Institute, Nanjing, China), according to the manufacturer's instructions. We added KCN (3 mmol/L) into mitochondrial lysates to inactivate residual Cu/ZnSOD and extracellular SOD before performing assays, resulting in the detection of only MnSOD activity. The enzymatic activity of MnSOD was expressed as a ratio of citrate synthase activity. Each sample was tested in triplicate.

**Figure 8:**
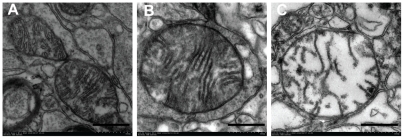
Representative electron micrographs of mitochondria (×100,000). Mitochondria were isolated from the swine frontal cortex at 24 h following ROSC. A: normal mitochondria with intact membrane cristae and a smooth matrix in the blank control group. B: slightly damaged mitochondria with basically normal cristae and a slightly damaged matrix in the hypothermia group. C: markedy deranged mitochondria with disrupted cristae and a damaged matrix in the normothermia group. Scale bar  = 500 µm.

### Assay for respiratory enzymes activity

Mitochondria were lysed using the animal tissue mitochondria lysis kit (GENMED, Shanghai, China), and the activities of Complex I and Complex III in the mitochondrial respiratory chain were measured using the animal mitochondrial respiratory chain complexes I and Complex III activities quantitative determination kit (GENMED, Shanghai, China). Complex I activity was calculated as the rotenone-specific activity subtracted from the total activity. The above was performed at 4°C. The activities of mitochondrial respiratory chain complexes were expressed as a ratio of citrate synthase activity. Each sample was tested in triplicate.

### Western blotting analysis of MnSOD and Nrf2

Cytoplasmic and nuclear protein fractions were prepared using NE-PER nuclear and cytoplasmic extraction reagents (Pierce Biotechnology, Inc, Rockford, IL, USA) based on the manufacturer's instruction. Protein concentration of cell lysates was determined using the Bradford method. Aliquots of protein (30 µg) of cytoplasmic, nuclear or mitochondrial fractions were separated in 10% SDS-PAGE and transferred to nitrocellulose membrane. After blocking with 5% nonfat milk in PBS containing 0.2% Tween-20, membranes were incubated at 4°C overnight with primary antibody, including rabbit polyclonal anti-Nrf2 (1∶300; Abcam, Cambridge, MA, USA), rabbit polyclonal anti-MnSOD (1∶500; StressGen, Assay Designs, Inc., Ann Arbor, MI, USA), mouse polyclonal anti-GAPDH (glyceraldehyde-3-phosphate dehydrogenase) (1∶1000; Abcam, Cambridge, MA, USA), mouse monoclonal anti-Lamin B2 (1∶300; Pierce Biotechnology, Inc, Rockford, IL, USA), rabbit polyclonal anti-VDAC1 (voltage-dependent anion channel)/Porin (1∶600; Abcam, Cambridge, MA, USA) and mouse polyclonal anti-β-actin (1∶1000; Sigma-Aldrich, St. Louis, MO, USA). Membranes were then incubated with horseradish peroxidase-conjugated secondary antibodies (Sigma-Aldrich, St. Louis, MO, USA) for 2h. ECL reagent (GE Healthcare, Piscataway, NJ, USA) was used for protein detection. We measured optical densities of the immunoreactive bands using ImageJ software (National Institutes of Health, Bethesda, MD, USA). MnSOD, and cytoplasmic, nuclear and total Nrf2 protein levels were normalized to Porin, GAPDH, Lamin B2 and β-actin, respectively, and presented as a ratio.

### Real-time RT-PCR for expression of MnSOD and Nrf2 mRNAs

We extracted total RNA from frozen swine frontal cortex samples with Trizol reagent (Invitrogen, Carlsbad, CA, USA), followed by reverse transcription to generate first-strand cDNAs using the QuantiTec reverse transcription kit (Tiangen Biotech, Beijing, China), according to the manufacturer's instructions. As the swine cDNA sequences for Nrf2 were not available in GenBank, we performed RT-PCR to obtain its partial coding sequence in advance, using primer pairs (Nrf2, F-5′-TATGGAGACACACTGCTTGG-3′and R-5′- GCATTTCACATCACAGTAGG-3′) designed by OLIGO 4.0 program based on the multialignment of ortholog sequences known in humans. The partial sequence of Nrf2 is available on request. Subsequently, we determined the expression of Nrf2 and MnSOD mRNAs by real-time RT-PCR. The primer sequences for MnSOD (Sus scrofa) were F-5′- CTGGAAGCCATCAAACGC-3′ and R-5′- TGAAACCGAGCCAACCC-3′, and the primer sequences for the endogenous reference β-actin (Sus scrofa) were F-5′- TGGGTATGGGTCAGAAAG-3′ and R-5′- CTCGTTGTAGAAGGTGTG-3′. We used the Quant script RT Kit (Tiangen Biotech, Beijing, China) for real-time RT-PCR analysis. The cycle threshold (Ct) values of the interested genes were first normalized with β-actin from the same sample, and then the relative differences between the groups were calculated and expressed as relative increases, setting the blank control group as 1. Each sample was tested in triplicate.

### Histopathology in the cortex and hippocampus

Tissue blocks (0.5 cm thick) from the cortex and hippocampus were embedded in paraffin, sliced into 6 µm thick sections and stained with hematoxylin and eosin (HE). In each microscopic field at a magnification of ×400, the percentage of damaged neurons was determined under a light microscope (Shanghai zousun optical instrument Co., Ltd, Shanghai, China) by an experienced doctor from the department of pathology in a blind fashion. The criteria for neuronal cytopathology included an eosinophilic cytoplasm, cytoplasmic vacuolation, perikaryal shrinkage, and nuclear pyknosis [24]. Three sections at least 15 µm apart were examined for each pig and the mean value was taken.

### Electron microscopy

Approximately 1 mm thick of cerebral cortex was sliced on ice, immediately fixed overnight at 4°C with a 2.5% (v/v) glutaraldehyde and 2.0% (w/v) paraformaldehyde, postfixed with 1% (v/v) osmic acid for 2 h, and then dehydrated and embedded with Epon812. The semi-thin sections were cut and stained with 1.0 % (w/v) toluidine blue and observed under a light microscope (Shanghai zousun optical instrument Co., Ltd, Shanghai, China). Ultra-thin sections (approximately 40–50 nm) were cut, double stained with 2.0% (w/v) uranyl acetate and 2.0% (w/v) lead citrate, and finally observed blindly under the HITACHI H-600 transmission electron microscope (Hitachi Scientific Instruments, Mountain View, CA, USA).

To quantitatively assess the ultrastructural changes of mitochondria, we used the morphometric analysis described previously [21] with minor modifications. Briefly, at least four different electron microscopic micrographs representing different areas in an ultra-thin section from each pig were selected, and the mean of these scores from the same ultra-thin section was regarded as the score of a pig. The criteria for scoring were as follows: a score from two to zero was given, where two was normal/not damaged, one was influenced, and zero was bad. Both the matrix and the membrane cristae were evaluated. Mitochondria displaying a homogenous density in the matrix were scored as two. Well-defined, tightly connected membrane cristae of mitochondria were also scored as two. Thus, a maximum score of four and a minimum score of zero could be obtained for mitochondria.

### Neurological deficit scores (NDS)

We adopted NDS to evaluate the outcome of neurologic function in pigs at 24 and 72 h following ROSC. NDS included the levels of consciousness, motor and sensory function, respiratory pattern, as well as behavior. The scores from each category were summed. A minimum score of zero indicated that animals were normal, whereas a total score of 400 represented brain death [25]. Investigators were blinded to the pigs' respective treatments.

### Statistical analysis

All data are presented as mean ± standard deviation (SD) except for NDS and ultrastructural scoring of mitochondria, which are expressed as median and range. Three-way ANOVA (factors: temperature, time, and gender) was used to assess overall differences among groups for each of the variables, followed by Bonferroni test for multiple comparisons. Levene's test for equality of variance was used to indicate the multiple-comparison procedure to be used. Survival rates were compared using Fisher's exact test. Ultrastructural scoring of mitochondria was statistically analyzed using nonparametric Mann-Whitney U-test. Differences were considered statistically significant when the *p* value <0.05. Analysis was performed using the software package SPSS 16.0 (SPSS Inc., Chicago, Illinois, USA).

## Results

### Physiologic, hemodynamic and resuscitation data

There was no significant difference in the number of male or female pigs in each group (Figure 1). At baseline, 24 h or 72 h after ROSC, weight, temperature, arterial lactate and PaO_2_, as well as hemodynamic parameters, were similar for pigs in all groups. Twelve hours after ROSC, heart rate and cardiac output (CO) were significantly decreased in the HT group compared with the NT group (*p*<0.01). CPR time was not statistically different between the NT and HT groups (3.3±1.2 *vs*. 3.4±1.3 min, respectively; Table 1).

### Survival rate and neurological outcome

We successfully resuscitated 32 pigs. Following ROSC, 6 out of 8 pigs in the NT-24 h, 7 out of 8 in the HT-24 h, 5 out of 8 in the NT-72 h, and 7 out of 8 in the NT-72 h survived (Figure 1). Although there was a trend towards higher survival rates at 24 and 72 h after ROSC in the HT group compared with the NT group (87.5% *vs.* 75.0%, 87.5% *vs*. 62.5%, respectively), the difference did not reach statistical significance (*p* = 0.60, *p* = 0.32, respectively). Seven pigs (3 females, 4 males) died due to hemodynamic instability during the post-resuscitation period. In the NT or HT group, the differences in total survival rate between the females and the males were not statistically significant (*p* = 0.68, *p* = 0.48, respectively).

We found that the NDS were significantly lower in the HT group when compared with the NT group at 24 h and 72 h after ROSC (*p*<0.01; Figure 2), indicating mild hypothermia could improve neurological outcome. In addition, the NDS for female pigs were slightly lower than for male pigs at 24 h and 72 h after ROSC in the NT or HT group, but this difference was not statistically significant (all *P*>0.05).

### Effect of mild hypothermia on mitochondrial lipid and protein peroxidation

To evaluate oxidative stress in mitochondria isolated from the pig cerebral cortex at 24 h and 72 h after ROSC and determine the effect of mild hypothermia, we first examined the extent of lipid and protein peroxidation in mitochondria. MDA and protein carbonyls were significantly increased in brain mitochondria from normothermic and mild hypothermic pigs at 24 h after ROSC when compared to blank control pigs (*p*<0.01), whereas the increase was attenuated in the HT group when compared with the NT group (*p*<0.01; Figures 3A and 3B). Seventy-two hours after ROSC, MDA and protein carbonyl levels almost returned to baseline levels, but there was no difference between the NT and HT groups (Figures 3A and 3B). Moreover, in the NT or HT group MDA and protein carbonyl levels for the female pigs showed a slight decrease when compared to the male pigs (all *P*>0.05).

### Effect of mild hypothermia on respiratory enzymes activity

Complex I and Complex III activity are significantly decreased at 24 h after ROSC (*p*<0.01), and were restored at 72 h after ROSC (Figures 4A and 4B). However, mild hypothermia attenuated this decrease (*p*<0.01), indicating its ability to reduce impairment of respiratory enzymes and thus reduce the production of ROS. In the NT or HT group, the differences in Complex I and Complex III activity between females and males all had no statistical significance (all *P*>0.05).

### Effects of mild hypothermia on MnSOD activity, and MnSOD mRNA and protein expressions

To determine the effects of mild hypothermia on the activity of MnSOD, and on the expression of MnSOD mRNA and protein in the pig cerebral frontal cortex at 24 h and 72 h following ROSC, we examined enzymatic activity spectrophotometrically, protein levels using western blotting analysis and mRNA levels using real-time PCR. We observed a significant increase in MnSOD activity (*p*<0.01; Figure 4C), and a consistent increase in MnSOD protein and mRNA expression caused by global brain I/R in the NT-24 h group when compared with the BC group. Interestingly, high MnSOD activity, upregulated expression of MnSOD protein and mRNA in the HT group was observed when compared with the NT group (Figures 4C, 5A and 6A). We also found, at 72 h following ROSC, that MnSOD activity, and MnSOD protein and mRNA expression, were slightly increased in the NT group, whereas there were no significant difference between the HT and NT groups (Figures 4C, 5A and 6A).

Though we found that in the NT or HT group MnSOD activity for female pigs was slightly high when compared to male pigs at 24 h and 72 h after ROSC, this difference did not have statistical significance (all *P*>0.05). In each group, no significant gender difference was found in MnSOD mRNA levels (all *P*>0.05).

### Effects of mild hypothermia on Nrf2 protein and mRNA expressions, and Nrf2 nuclear translocation

As mentioned before, Nrf2 plays a central role in maintaining redox balance. The Nrf2-dependent cytoprotective pathway has been shown to induce gene expression of antioxidant enzymes including SOD. We therefore examined the effects of mild hypothermia on Nrf2 nuclear translocation in the pig cerebral frontal cortex. Western blotting showed that Nrf2 protein in the nuclear fraction increased, whereas it decreased in the cytosolic fraction at 24 h following ROSC in the NT group when compared with the BC group (Figure 5B), indicating translocation of Nrf2 from the cytosol to the nucleus. In addition, we found that Nrf2 nuclear translocation was significantly elevated in the HT group when compared with the NT group (Figure 5B).

To investigate the translational regulation of Nrf2 triggered by oxidative stress, we next examined the expression of Nrf2 mRNA in the cerebral frontal cortex in our pig cardiac arrest model using real-time PCR. The results showed a significant increase in the expression of Nrf2 mRNA in the NT group when compared with the BC group, and higher Nrf2 mRNA expression in the HT group when compared with the NT group (Figure 6B). Also, we found that the increase in Nrf2 mRNA expression was consistent with the change of total Nrf2 protein level.

Moreover, we found, at 72 h following ROSC, that Nrf2 protein in the nuclear fraction and Nrf2 mRNA were slightly increased in the NT group, whereas there were no significant differences between the HT and NT groups (Figures 5B and 6B).

In each group, no significant gender difference was found in Nrf2 mRNA levels and optical density of bands for cytosolic and nuclear Nrf2 (all *P*>0.05).

### Effect of mild hypothermia on histopathology in the cortex and hippocampus

HE staining showed that, compared to normothermia, treatment with mild hypothermia significantly decreased the number of damaged neurons (Figure 7) in the precentral gyrus of the frontal lobe (45.3%±4.2% *vs* 28.3%±2.7%, *P*<0.01) and in the CA1 area of the hippocampus (63.4%±5.2% *vs* 35.3%±2.9%, *P*<0.01) at 24 h following ROSC. Furthermore, in each group no significant gender difference in the number of damaged neurons in the precentral gyrus of the frontal lobe and the CA1 area of the hippocampus was observed, however, the female pigs showed a slightly decreased number of damaged neurons when compared to the male pigs (all *P*>0.05).

### Effect of mild hypothermia on mitochondrial morphology determined by electron microscopy

At 24 h following ROSC, ultrastructural damage of mitochondria in the cerebral cortex was reduced in the HT group when compared to the NT group (Figure 8) with a significantly elevated score (3.2 [2.5–4.0] *vs*1.5 [1.0–2.0], *P* = 0.001). There was no significant difference between female and male pigs in each group (all *P*>0.05).

## Discussion

Our experiments demonstrate that after treatment with whole-body mild hypothermia, (1) neurological outcome is improved, (2) mitochondrial oxidative stress in the cerebral cortex is attenuated even at 24 h following ROSC, (3) impairment of mitochondrial respiratory chain enzymes (Complex I and Complex III) is reduced, (4)increased activity of MnSOD is elevated, (5) upregulated expressions of MnSOD protein and mRNA are enhanced and (6) activated nuclear factor Nrf2 is enhanced at 24 h and 72 h following ROSC.

To mimic the clinical application of mild hypothermia in patients with cardiac arrest [7], by means of the CoolGard 3000 system, we intravascularly induced cooling, maintained mild hypothermia (32°C–34°C ) for 12 h, and then actively rewarmed (0.5°C/h) in a well established swine cardiac arrest model induced by VF [26], [27], [28], [29], [30], [31], [32], [33]. The results showed a significantly lower NDS in the HT group when compared with the NT group at 24 h or 72 h after ROSC, indicating mild hypothermia could improve neurological outcome. This is consistent with previous evidence from animals and humans [1], [4], [6], [7], [8]. There was a trend towards higher survival rates presented at 24 h and 72 h after ROSC, nevertheless the difference did not reach statistical significance. Morphologically, we also observed that mild hypothermia alleviated microscopic and ultrastructural changes in the cerebral cortex caused by global I/R at 24 h after ROSC (Figure 7 and 8).

The effect of hypothermia on oxidative stress is controversial. Camara et al. found that hypothermia moderately enhanced superoxide anion (O_2_^−^) generation by mitochondria, and markedly slowed O_2_^−^ dismutation in the guinea pig isolated perfused heart [34], suggesting that hypothermia may increase oxidative stress. However, Lei et al. reported that mild hypothermia decreased lipid peroxidation in the dog cerebral cortex 2 h following ROSC [35]. In this study, we demonstrated that whole-body mild hypothermia attenuated mitochondrial oxidative stress in the cerebral cortex even at 24 h following ROSC, consistent with the result of Lei et al. The reasons for this discrepancy are thought to include different animal models, and different depth of hypothermia. Cold may be a sort of stress to cells, and thus, lower temperatures could enhance oxidative stress. In the above study by Camara et al., the magnitude of ROS production was found to be inversely proportional to the temperature change [34]. Moreover, most enzyme activities decreases by 50% for every 10°C fall in temperature [36]. It is likely that lower temperatures not only increase ROS generation but also decrease removal of ROS due to the reduced activity of enzymes responsible for scavenging ROS.

Any alteration in the balance between the generation and removal of ROS is considered oxidative stress. To determine the mechanisms underlying the attenuated oxidative stress by mild hypothermia, we further investigate the generation and removal of ROS.

Possibly up to 90% of intracellular ROS are generated in the mitochondrial electron transport chain (namely complexes I and III) where electron leakage normally occurs [37]. After ROSC, especially in the early stage of post-ROSC, mitochondrial oxidative phosphorylation is slowed down, resulting in reduced generation of ATP, and thereby electrons are diverted to the Q-cycle through the electron transport chain, generating excessive ROS. Mitochondrial electron transport chain *per se* could be impaired by ROS at the same time of generating ROS. In this experiment, we demonstrated that the activity of Complex I and Complex III was significantly reduced in the cerebral cortex mitochondria from normothermic pigs when compared to blank control pigs not subjected to I/R injury, consistent with previous studies [5], [38]. Moreover, we found that the decreased activities of Complex I and Complex III by I/R was significantly improved after treatment with mild hypothermia, indicating mild hypothermia could reduce impairment of respiratory enzymes. It was reported that the rate of mitochondrial ROS generation is inversely proportional to the rate of oxidative phosphorylation, increasing when activity of mitochondrial respiratory enzymes are inhibited [37], [39]. We thereby speculate that the improved activity of mitochondrial respiratory enzymes could attenuate the overproduction of ROS even at 24 h following ROSC.

MnSOD is a nuclear encoded primary antioxidant enzyme located exclusively in the matrix of the mitochondrion [40] and is therefore a pivotal part of the antioxidant defense involved in protecting against oxidative injury to mitochondria [41]. We found a significant increase in MnSOD activity caused by global brain I/R in the NT-24 h group. To clarify the mechanisms underlying the increased MnSOD activity observed in the cerebral cortex from pigs following ROSC, we further examined the change of the protein and mRNA levels of MnSOD, and found that the upregulation in MnSOD protein and mRNA levels are consistent with a higher activity of MnSOD in normothermic pigs, whereas MnSOD protein and mRNA levels were upregulated after rewarming in hypothermic pigs. Based on the presence of protein and lipid peroxidation, we speculate that the enhanced ROS generation could overwhelm the removal of ROS in the cerebral cortex from pigs following ROSC, and that the increased MnSOD activity and reduced ROS generation after treatment with mild hypothermia could lead to a decrease in oxidative stress, and thus protect against global brain I/R injury.

SOD is known to be regulated by Nrf2. Nrf2 is a basic leucine zipper (bZIP) transcription factor that serves as a central regulator of genes encoding a battery of antioxidant proteins and electrophile enzymes [10]. To our knowledge, it is found for the first time that there was a translocation of Nrf2 to the nucleus from the cytoplasm, which may result from oxidative stress, in the cerebral cortex from normothermic pigs following ROSC, and that this translocation of Nrf2 was consistent with an increase in MnSOD protein and mRNA expression. Also, we found that there was an increase in Nrf2 mRNA and protein expression in the cerebral cortex from normothermic pigs following ROSC. More importantly, these tendencies were enhanced after treatment with mild hypothermia.

Because of the difficulty in supplying inbred Chinese Wuzhishan minipigs, we did not choose pigs of the same sex. In the present study, analysis by gender showed that no significant gender differences were found for post-resuscitative brain damage and on mild hypothermic effects, although female pigs had a slightly better outcome. However, increasing evidence demonstrates that sexual hormonal differences due to sex dimorphism may be implicated in the outcome of cerebral ischemia; in particular, the neuroprotective effects of estrogen have been widely documented in animals and humans [42], [43], [44], [45]. There are several factors for this discrepancy. First, in female animals, the estrous cycle affects the outcome of ischemic brain injury [46]. However, in our study, all female pigs may have had low endogenous estrogen levels because they were not in a state of estrus. Second, most of the pigs (31/37) were sexually immature due to their age (∼4 months old). Finally, the small sample size of male and female pigs in each group may have failed to test for potential differences. Although there was no significant difference in gender among the control, normothermic and hypothermic groups, gender difference may be a potentially confounding factor.

However, our work has some limitations. First, to mimic the unbroken process of whole-body mild hypothermia clinically applied to cardiac arrest patients [7], we only aimed to the effects after rewarming rather than during mild hypothermia. Oxidative stress in mitochondria occur mainly in the early stages of reperfusion, whereas it may last for dozens of hours even several days due to the involvement of a myriad of secondary injury after ROSC. Second, we did not concern with cytosolic copper-zinc SOD (Cu/ZnSOD) and extracellular SOD (EsSOD), as well as other antioxidants and antioxidant enzymes regulated by Nrf2, e.g. GSH, catalase, which contribute to oxidative stress in mitochondria, yet it is a early episode of ROS removal and a key reaction that MnSOD catalyzes the dismutation of the superoxide anion into oxygen and hydrogen peroxide [41].

In conclusion, our work has demonstrated that mitochondrial oxidative stress of cerebral cortex is attenuated after treatment with whole-body mild hypothermia even at 24 h following ROSC, which may be one of the mechanisms underlying neurologic protection. It appears that the effect of mild hypothermia on mitochondrial oxidative stress is achieved, at least in part, by its ability to reduce impairment of respiratory chain enzymes. Also, we have demonstrated that the increased activity of MnSOD, and the upregulated expression MnSOD protein and mRNA are further enhanced after treatment with whole-body mild hypothermia even at 24 h and 72 h following ROSC via the activation of nuclear factor Nrf2, which may contribute to the attenuation of mitochondrial oxidative stress.
